# Survival of patients with head and neck cancer with metachronous multiple primary tumors is surprisingly favorable

**DOI:** 10.1002/hed.25595

**Published:** 2019-01-16

**Authors:** Oisín Bugter, Dirk L.P. van Iwaarden, Emilie A.C. Dronkers, Martine J. de Herdt, Marjan H. Wieringa, Gerda M. Verduijn, Marc A.M. Mureau, Ivo ten Hove, Esther van Meerten, José A. Hardillo, Robert J. Baatenburg de Jong

**Affiliations:** ^1^ Department of Otorhinolaryngology and Head and Neck Surgery Erasmus MC Cancer Institute Rotterdam The Netherlands; ^2^ Department of Radiation Oncology Erasmus MC Cancer Institute Rotterdam The Netherlands; ^3^ Department of Plastic and Reconstructive Surgery Erasmus MC Cancer Institute Rotterdam The Netherlands; ^4^ Department of Oral & Maxillofacial Surgery, Special Dental Care, and Orthodontics Erasmus MC Cancer Institute Rotterdam The Netherlands; ^5^ Department of Medical Oncology Erasmus MC Cancer Institute Rotterdam The Netherlands

**Keywords:** esophageal cancer, head and neck cancer, incidence, multiple primary tumors, survival analysis

## Abstract

**Background:**

The objectives of this study are to determine the incidence and survival rate of patients with head and neck squamous cell carcinoma (HNSCC) with multiple primary tumors (MPT) in the HN‐region, lung, or esophagus.

**Methods:**

Patient and tumor specific data of 1372 patients with HNSCC were collected from both the national cancer registry and patient records to ensure high‐quality double‐checked data.

**Results:**

The total incidence of MPTs in the HN‐region, lung, and esophagus in patients with HNSCC was 11% (149/1372). Patients with lung MPTs and esophageal MPTs had a significant worse 5‐year survival than patients with HN‐MPTs (29%, 14%, and 67%, respectively, *P* < 0.001). The 5‐year survival rate for synchronous HN MPTs was only 25%, whereas it was surprisingly high for patients with metachronous HN MPT (85%, *P* < 0.001).

**Conclusions:**

One of 10 patients with HNSCC develop MPTs in the HN‐region, lung, or esophagus. The 5‐year survival of patients with metachronous HN MPTs was surprisingly favorable.

## 1 INTRODUCTION

Head and neck cancer (lip, oral cavity, nasopharynx, oropharynx, hypopharynx, and larynx) has an increasing incidence with 686 000 new cases and 404 000 associated mortalities worldwide in 2012.^1^ The majority of head and neck tumors are squamous cell carcinoma (HNSCC).^2^ Due to advances in surgical and radiotherapy and chemotherapy techniques, the 5‐year survival of patients with HNSCC has improved from 55% in 1992‐1996 to 66% in 2002‐2006.^3^ This relatively low survival rate could be explained by high tumor stages at diagnosis, patient delay before diagnosis, and a high incidence of tumor recurrence.^4^, ^5^, ^6^, ^7^ Another important factor affecting survival might be the development of multiple primary tumors (MPTs) in the head and neck region (HN‐region) but also in associated organs such as the lung and esophagus.^5^, ^8^

MPTs are squamous cell tumors, which develop at or after diagnosis of the index tumor.^9^, ^10^ Patients with second (SPT), third, fourth, or even more primary tumors are defined as patients with MPTs. MPTs are not the same as a residual/recurrent tumors, which occur at the same site as the index tumor. For patients with an index HNSCC, MPTs most frequently occur in the HN‐region, lung, or esophagus.^5^, ^11^

The concept that explains the occurrence of MPTs is field cancerization (FC). FC implies that tumors do not arise as an isolated tumor but occur in a field of pre‐neoplastic squamous cells that have an anaplastic tendency. This tendency gives rise to a multifocal development of tumors at various rates within the field.^12^ For patients with HNSCC, this FC is thought to extend as far as the lung and esophagus.^13^ There are several theories that explain FC. The first is the polyclonal theory, which states that multiple precursor fields arise under the influence of carcinogenic agents.^12^ The other theories are based on monoclonal concepts with a spread of dysplastic cells, which give rise to new fields in which MPTs may develop.^14^

The incidence of MPTs in patients with HNSCC is reported to range from 9.4% to 14.4%.^5^ Most second primary tumors (SPTs) occur in the HN‐region (40%‐59%), lung (31%‐37%), and esophagus (9%‐44%).^5^ The overall survival rate of patients who develop MPTs is lower than the survival of patients with only a single primary tumor.^6^ A major decrease of 5‐year overall survival rates from 69% to 32% has been reported for patients with metachronous MPTs compared to patients without MPTs.^15^, ^16^ It has even been suggested that MPTs could have a worse effect on the survival of patients with HNSCC than residual/recurrent tumors of the index tumor.^5^, ^17^

In the literature, there is limited information available on the incidence and impact of MPTs on the survival of patients with HNSCC with a white ethnicity. Most studies on this topic have been performed in Asia; therefore, the results may not be generalizable for patients with HNSCC in Western countries, because tumor incidences vary widely.^18^ Subsequently, incidence and survival rates of patients with MPTs could be underestimated or overestimated. Also, cohorts that include a large number of patients are scarce.

The main objective of this study is to describe the incidence of MPTs in a large Dutch cohort of patients with HNSCC. The second objective is to analyze the effect of MPTs on the survival rates of patients with HNSCC.

## 2 PATIENTS AND METHODS

This article was written according to the STROBE guidelines for reporting observational studies.^19^ It was approved by the Medical Ethics Committee of the Erasmus MC (MEC‐2016‐751).

### 2.1 Patients

Patients were selected from the Rotterdam Oncology Documentary (RONCDOC), which is a database that compromises all patients with head and neck cancer treated at the Erasmus MC Cancer Institute since 1995. We included all 1372 patients who had been diagnosed with an HNSCC (lip, oral cavity, nasopharynx, oropharynx, hypopharynx, larynx, and sinonasal cavity) as index tumor between January 1, 2008 and December 31, 2011. The final date of follow‐up was August 14, 2017. No patients were excluded. Patients were divided into three groups: patients who developed a second primary tumor in the (a) HN‐region, (b) lung, or (c) esophagus.

### 2.2 Data collection

Patient, tumor, and therapy data were acquired from the Netherlands Comprehensive Cancer Organization (a national cancer registry in which all histologically proven cancers in the Netherlands are registered—irrespective of the hospital where the cancer is diagnosed) and merged with data from the patient records of the Erasmus MC Cancer Institute. Subsequently, the data were manually checked for each patient using available data from the patient records. If there was any doubt about the validity of the data collected, the patient was discussed by the research staff until a consensus was reached. A log was kept in which the inclusion of patients was recorded. This leads to a high degree of classification accuracy and low risk of selection bias. The following data were collected: date of birth and death, last follow‐up date, comorbidity, prior malignancies, tobacco and alcohol consumption, body mass index (BMI), clinical and histopathologic TNM and tumor stage, type and intention of therapy, and location and time to occurrence of MPTs.

Multiple primary tumors were defined according to the Warren & Gates and Hong et al. criteria, which state that the MPT (a) must be diagnosed as malignant on histologic examination, (b) must be histologically distinct from the index tumor and thus not a metastasis, (c) has to be at least 2 cm from the site of the index tumor or the tumor has to occur > 3 years after the diagnoses of the index tumor.^9,10^ Patients with second, third, fourth, or even more primary tumors (> 1 primary tumor) were identified as patients with MPTs. An SPT is thus a first MPT. An MPT was defined as synchronous if the tumor developed < 6 months after the diagnosis of the index tumor and as metachronous if it developed after ≥ 6 months.

A distinct differentiation should be made between MPTs and residual/recurrent tumors, which occur at the same site and share the same histopathology as the index tumor. Residual tumors develop < 6 months after the index tumor and recurrent tumors ≥ 6 months, but < 3 years. A tumor developed at the same site as and ≥ 3 years after the index tumor was considered to be an MPT.

Comorbidities were scored with the Adult Comorbidity Evaluation‐27.^20^ The intention of therapy was scored as curative or palliative based on the Dutch guidelines for the treatment of HNSCC, lung carcinoma, and esophagus carcinoma.^21^ Height and weight were used to calculate the BMI. Patients were categorized as underweight (BMI < 18.5), normal weight (BMI 18.5‐24.9), overweight (BMI 25‐29.9), and obese (BMI ≥ 30). Tobacco and alcohol use was registered as current‐, previous‐ or non‐smoker/drinker. For tobacco use, the number of pack‐years was registered and for alcohol use the number of units per week was registered.

### 2.3 Statistical analysis

If quantitative variables were normally distributed, the results are expressed as mean values and SD; otherwise median and interquartile range (IQR) are used. Categorical data are reported as frequencies and percentages, and differences between groups were analyzed using the chi‐squared test. A Kaplan‐Meier survival analysis was used for survival analyses and the log‐rank test to compare the survival distributions of two groups of patients. The 5‐year survival from the date of diagnosis of the index tumor was analyzed and, additionally, the 3‐year survival rate from the date of diagnosis of the SPT was analyzed. The survival rate was analyzed separately for patients with synchronous a metachronous SPTs. A complete case analysis was used to handle missing data. However, all data on the outcomes of interest (incidence and survival) were complete. Statistical analysis was performed using SPSS version 21.0 (IBM Corp., Armonk, New York).

## 3 RESULTS

### 3.1 General patient and index tumor characteristics

A total of 149 patients with multiple primary tumors and an HNSCC as index tumor were identified in our cohort. Their baseline characteristics are shown in detail in Table 1. The mean duration of follow‐up was 51.9 months (SD 27.9). One hundred eleven patients (74.5%) were men and their mean age was 63.1 years (SD 8.8). The majority of patients was a current smoker (114 [76.5%]). This group had a median number of 42.0 pack‐years (IQR 33.0‐58.8). The majority of patients was also current alcohol abusers (114 [76.5%]), who had a median alcohol consumption of 28 units per week (IQR 14‐42). There were 110 patients (73.8%) with mild to severe comorbidity.

**Table 1:** Patient characteristics

Number of patients	149
Follow‐up, mean (SD), mo	51.9 (27.9)
Male sex, n (%)	111 (74.5)
Age, mean (SD), y	63.1 (8.8)
Smoking status, n (%)/median PY (IQR)	
Current smoker	114 (76.5)/42 (33‐59)
Previous smoker	27 (18.1)/40 (25‐50)
Nonsmoker	7 (4.7)/0 (0–0)
Missing	1 (0.7)
Alcohol consumption, n (%)/median UPW (IQR)	
Current drinker	114 (76.5)/28 (14–42)
Previous drinker	22 (14.8)/28 (14–42)
Nondrinker	12 (8.0)/0 (0–0)
Missing	1 (0.7)
Comorbidity^a^, n (%)	
None	39 (26.2)
Mild	57 (38.2)
Moderate	31 (20.8)
Severe	22 (14.8)
Body mass index, n (%)	
Underweight (< 18.5)	10 (6.7)
Normal weight (18.5–24.9)	78 (52.3)
Overweight (25.0‐29.9)	47 (31.5)
Obese (≥ 30)	9 (6.2)
Missing	5 (3.3)

Abbreviations: PY, pack‐years; UPW, units per week.

a Comorbidity measured by Adult Comorbidity Evaluation 27.

Table 2 provides a detailed overview of the characteristics of the HNSCC index tumor. Most tumors were in the oral cavity (46 [30.9%]), followed by the oropharynx (40 [26.8%]) and the supraglottic region (28 [18.8%]. The tumor stage ranged from 0 (carcinoma in situ) to IV. Radiotherapy was the most frequently used therapy (52 patients [34.9%]), whereas 36 patients (24.2%) received surgery. Thirty‐two patients (21.5%) received surgery with adjuvant radiotherapy. Twenty patients (13.4%) received a combination of radiotherapy and chemotherapy. Recurrences of the index tumor occurred in 12 (8.1%) of the 149 cases. No residual tumors were detected.

**Table 2:** Characteristics of index tumor (n = 149)

Characteristics	n (%)
Tumor location	
Lip	2 (1.3)
Oral cavity	46 (30.9)
Oropharynx	40 (26.8)
Hypopharynx	11 (7.4)
Supraglottic	28 (18.8)
Glottis	18 (12.1)
Sinonasal cavity	4 (2.7)
Tumor stage	
0 (CIS)	6 (4.0)
I	39 (26.2)
II	34 (22.8)
III	26 (17.4)
IV	43 (28.9)
Missing	1 (0.7)
Therapy	
Surgery	36 (24.1)
Radiotherapy	52 (34.9)
Chemotherapy	0 (0.0)
Surgery + RT	32 (21.5)
RT + CT	20 (13.4)
Surgery + RT + CT	5 (3.4)
No therapy	4 (2.7)
Intention of the therapy	
Curative therapy	141 (94.6)
Palliative therapy	8 (5.4)
Residual tumors	0 (0.0)
Recurrent tumors	12 (8.1)

Abbreviations: CIS, carcinoma in situ; CT, chemotherapy; RT, radiotherapy.

### 3.2 MPT incidence and time to occurrence

**Figure 1:**
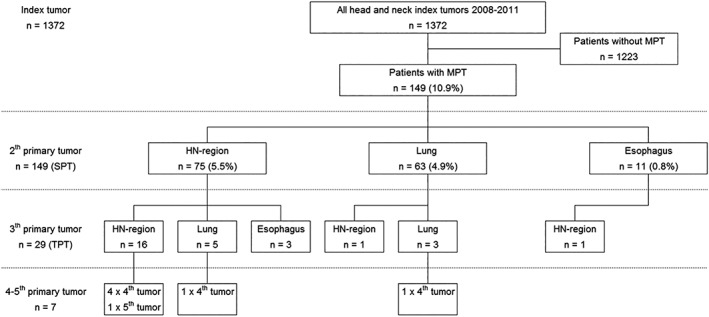
Flowchart presents the distribution of the multiple primary tumor development in patients with head and neck cancer. HN, head and neck; MPT, multiple primary tumor; SPT, second primary tumor; TPT, third primary tumor

Figure 1 presents the distribution of the MPT development during follow‐up. A total of 1372 patients with an HNSCC index tumor were diagnosed at the Erasmus MC Cancer Institute between 2008 and 2011. The total incidence of MPTs in patients with HNSCC was 10.9% (n = 149). The SPT of these patients was located in the HN‐region in 5.5% of the cases (n = 75), in the lung in 4.9% of the cases (n = 63), and in the esophagus in 0.8% (n = 11). Of these patients with an SPT, 19.5% (29/147) also developed a third primary tumor (TPT). Seven patients with TPTs (24.1%) even developed more than three primary tumors.

The median time to occurrence of all SPTs was 22.9 months (IQR 2.1‐47.4). The head and neck‐SPTs (HN‐SPTs) were synchronous in 23 cases (30.7%), with a median time to occurrence of 0.1 months (0.0‐0.9). Fifty‐two HN‐SPT cases (69.3%) were metachronous, with a median time to occurrence of 41.7 months (IQR 19.0‐58.0). The index tumors of patients with metachronous HN‐SPTs were more often advanced (stage III and IV) than synchronous HN‐SPTs (56.5% vs 26.9%). The SPTs in the lung were synchronous in 18 cases (28.6%) and had a median time to occurrence of 1.8 months (IQR 1.0‐2.7). Forty‐five lung‐SPTs (71.4%) occurred metachronously and had a median time to occurrence of 37.1 months (IQR 22.7‐55.0). Almost a quarter (n = 3) of the SPTs in the esophagus developed synchronously and the other 72.7% (n = 8) metachronously. The median time to occurrence from the index tumor to the TPT was 34.5 months (IQR 11.2‐60.0).

### 3.3 Survival analysis

**Figure 2:**
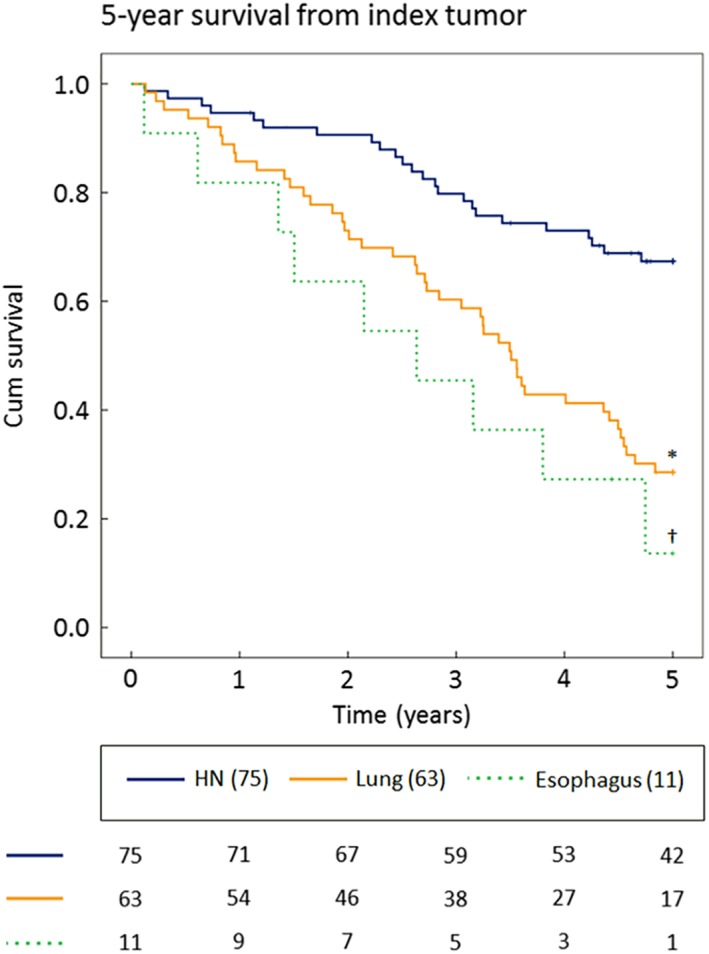
The 5‐year survival from the diagnosis of the HN index tumor for patients with second primary tumors in the HN‐region, the lung, and the esophagus. Numbers at the bottom of the figure represent patients at risk. *P < 0.001 compared to HN; † *P* < 0.001 compared to HN. *P*‐values calculated with log‐rank test. HN, head and neck [Color figure can be viewed at wileyonlinelibrary.com]

The survival of all 149 patients with MPTs was analyzed. Their overall 5‐year survival, measured from the occurrence of the index tumor, was 46.8%. The 5‐year survival of patients who developed an HN‐SPT (67.3%) was better than patients who had a lung‐SPT (28.6%, *P* < 0.001) or an esophageal‐SPT (13.6%, *P* < 0.001) (Figure 2).

**Figure 3:**
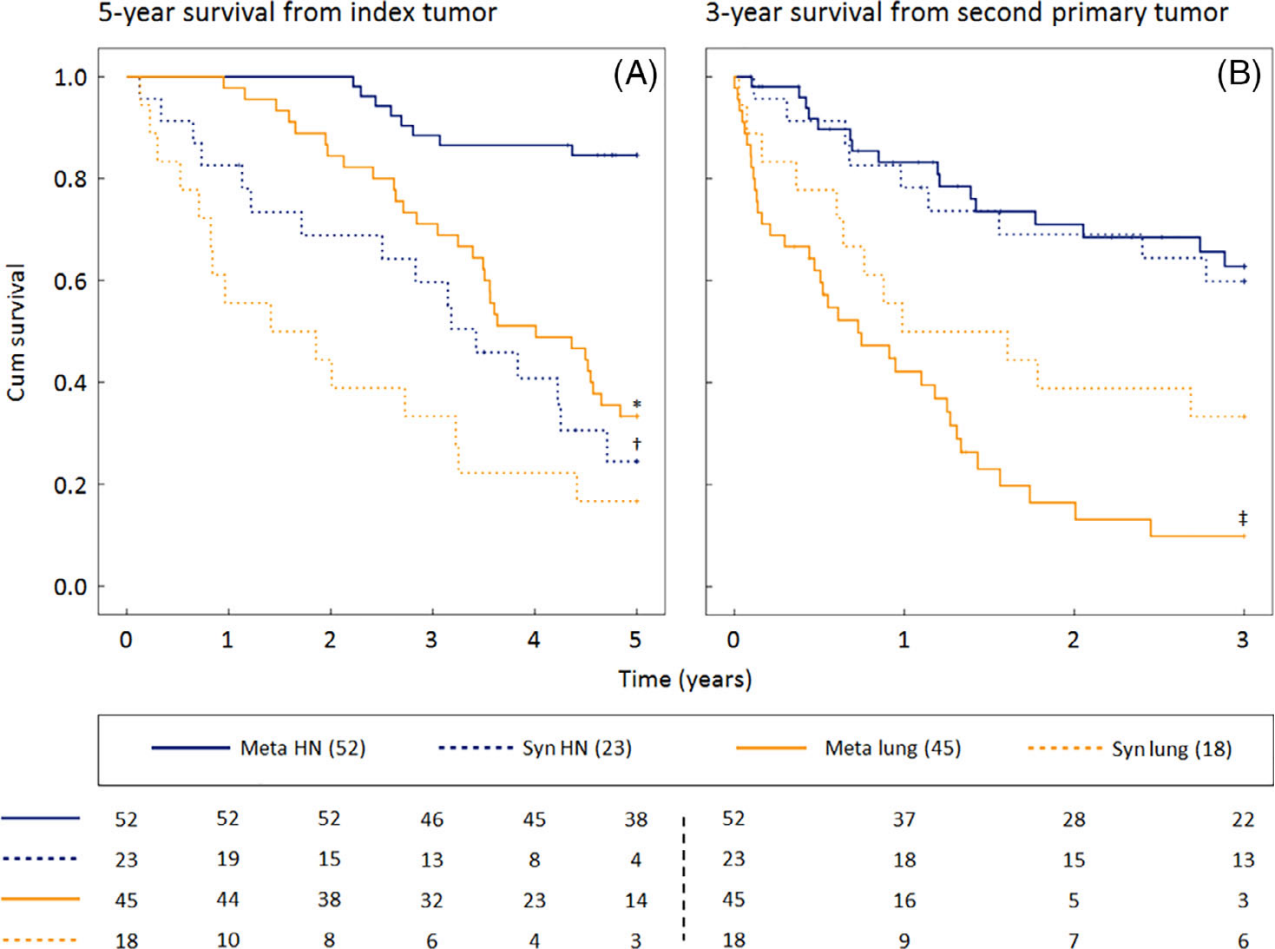
(A) The 5‐year survival from the diagnosis of the head and neck index tumor for patients with synchronous and metachronous second primary tumors in the head and neck region and the lung. (B) The 3‐year survival from the diagnosis of the second primary tumor for the same patients. Numbers at the bottom of the figure represent patients at risk. * *P* < 0.001 compared to meta HN, † *P* < 0.001 compared to meta HN, ‡ *p* = 0.048 compared to syn lung. *P*‐values calculated with log‐rank test. HN, head and neck; meta, metachronous; Syn, synchronous [Color figure can be viewed at wileyonlinelibrary.com]

Figure 3A shows that patients with synchronous HN‐SPTs had a worse 5‐year survival rate (24.5%) than patients with metachronous HN‐SPTs (84.6%, *P* < 0.001). The 5‐year survival of patients with a synchronous SPT in the lung (16.7%) was also worse than those with metachronous lung‐SPTs (33.3%, *P* = 0.003). Patients with metachronous lung‐SPTs had a lower 5‐year survival rate than patients with metachronous HN‐SPTs (*P* < 0.01). On the other hand, the survival of patients with a synchronous SPT in the lung and HN‐region was not significantly different (*P* = 0.19).

The median survival of patients with synchronous HN‐SPTs was 3.2 years (IQR 1.1‐4.4), whereas it was 6.1 years (IQR 4.8‐7.5) for metachronous cases. The median survival of patients with synchronous lung‐SPTs was 1.6 years (IQR 0.7‐3.5) and 4.0 years (IQR 2.7‐5.9) for patients with metachronous lung‐SPTs. Due to the limited number of patients with an esophageal‐SPT, we were not able to analyze differences between metachronous and synchronous SPTs in this group.

Figure 3B shows the 3‐year survival rate, measured from the moment the SPT was diagnosed. This was the same for patients with a synchronous (59.8%) and metachronous HN‐SPT (62.8%). A difference was seen in the group of patients with lung‐SPT. Patients with metachronous lung‐SPTs had a worse 3‐year survival rate (9.9%) than patients with synchronous lung‐SPTs (33.3%, *P* = 0.048). Metachronous lung‐SPTs were more often diagnosed in a high stage (stage III and IV) of development (34 [75.6%]) than synchronous lung‐SPTs (9 [50.0%], *P* = 0.049) and also more often than metachronous HN‐SPTs (21 [40.4%], *P* < 0.001).

## 4 DISCUSSION

This study showed that 1 of 10 patients with HNSCC develop at least one multiple primary tumor (MPT) in the HN‐region, lung, or esophagus. We acquired our results by using high‐quality, double‐checked data obtained from the national cancer registry and the patients records. Surprisingly, MPTs develop as frequently in lung (4.9%) as in the HN‐region (5.3%). The 5‐year survival rate of all patients was 47%. This is lower than the 66% stated in the literature.^3^, ^22^ Patients with MPTs that were synchronous or in the lung or esophagus had the worst survival. On the other hand, patients with metachronous HN‐SPTs had a surprisingly high 5‐year survival rate of 85%.

We showed that the 5‐year survival rate of patients with HNSCC with a synchronous HN‐SPT was significantly worse than patients with a metachronous HN‐SPT. This finding is similar to the results of two previous studies.^23^, ^24^ This could be explained by the higher percentage of high‐stage tumors (stage III and IV) in the synchronous HN‐SPT group (56%) compared to the metachronous HN‐SPT group (40%). However, this difference was not significantly different, *P* = 0.2. Another explanation is that the development of a synchronous MPT in the HN‐region limits the treatment options of the index tumor. Panosetti et al. showed that treatment protocols of the index tumor need to be adjusted when a synchronous SPT is diagnosed.^23^ The treatment strategy had to be adjusted in 50% of patients with HNSCC with a synchronous HN‐SPT. Subsequently, this adjustment caused a decline in the 5‐year survival rate from 18% to 8%.

The location of the SPT was also of significant influence on the survival. Although the incidence of SPTs in the HN‐region and lung were almost the same in the present study, the 5‐year survival of patients who developed an SPT in the lung (29%) was significantly worse than that of the patients with HN‐SPTs (67%). The survival rate of patients with esophageal‐SPTs was even lower (14%). These findings are in line with the results of other studies.^15^, ^25^

Interestingly, the 3‐year survival rate, measured from the occurrence of the SPT, was the same for patients with synchronous and metachronous HN‐SPTs (61% vs 63%). On the other hand, it was significantly lower for patients with lung‐SPTs: 33% for synchronous and 10% for metachronous MPTs. The difference between the 5‐year survival (from the index tumor) and 3‐year survival (from the SPT) of metachronous SPTs could be explained by the long median time to occurrence. This indicates that synchronous and metachronous SPTs have a similar mortality and that the time to occurrence of an SPT is what dictates patient survival. The first 6 months after the index tumor are important for the prediction of survival of an individual patient. Patients who developed an SPT within this period (synchronous) have a significant worse 5‐year survival rate, measured from the index tumor, than patients who stayed free of an SPT for the first 6 months (metachronous).

The majority of metachronous lung‐SPTs were diagnosed in stage III or IV (76%). This could be an explanation of the lower survival rate in this group compared to patients with synchronous lung‐SPTs or metachronous HN‐SPTs. Many patients with high‐stage lung tumors are incurable, and if treatment is available, it often induces severe comorbidity.^26^ To our knowledge, all current follow‐up protocols for patients with HNSCC lack an active screening for MPTs in the lung, despite the evident negative effect of lung‐MPTs on patient survival and the similar incidence as HN‐MPTs. Screening for lung‐MPTs could be considered because of the low survival rate of affected patients and the high percentage of high‐stage lung‐MPTs.^24^, ^27^

Several studies also advocate the use of surveillance and screening for esophageal‐MPTs.^28^ A French multicentered study investigated the use of endoscopy of the esophagus in the work‐up of patients with HNSCC to screen for MPTs. They found an eight‐times higher percentage of 6.8% esophageal carcinoma and high‐grade dysplasia than the 0.8% in our study.^29^ A study by De Vries et al. also showed high percentages of esophageal‐MPTs in a cohort of Dutch patients with HNSCC.^8^ Several Asian studies have even shown esophageal‐MPT incidences of up to 41%.^30^, ^31^, ^32^, ^33^ Therefore, we believe our incidence of esophageal‐MPTs is an underestimation of the actual incidence. This discrepancy between the literature and our findings could indicate that many esophageal‐MPTs are never diagnosed, despite the fact that diagnosis of early stage esophageal‐MPTs could improve the outcome of patients with HNSCC.^34^ It is even suggested that early esophageal‐MPT diagnosis and treatment could give these patients a similar prognosis as patients who did not developed an esophageal‐MPT.^35^ These findings suggest that endoscopic screening for esophageal‐MPTs in the workup of patients with HNSCC might cause a health benefit.

We showed an increasing risk to develop an MPT in patients who already have an MPT. The incidence increased from 11% for an SPT in patients with an HSNCC index tumor up to 24% for a fourth primary tumor in patients with three primary tumors. These findings are in line with the multifocal development of tumors within a precursor field, stated by the FC theory.^12^ Other literature showed that a continuous exposure to carcinogenic agents like smoking and alcohol and possibly radiotherapy treatment also increases the risk to develop an MPT.^36^ The increasing incidence and the FC theory combined give rise to the question whether patients with MPT can be completely cured.

No residual tumors were detected in this cohort, and the rate of recurrent tumors was 8.1%. This is relatively low, compared to a recent review that reported local residual/recurrence rates varying from 10% to 50%, depending on the location and stage of the primary tumor.^11^ This could be explained by the fact that all our patients have MPTs. Lester et al. stated that 85% of all recurrences appear after 13‐31 months.^37^ In comparison, the median time to occurrence of all SPTs in the present study was 23 months (IQR 2‐47). This could mean that a selection of our patients might have died as a consequence of an MPT before a recurrence could have developed. It could also indicate that less recurrences were diagnosed because diagnostics and treatment were focused on the MPT.

There are limitations to the present study that might have had an influence on the results we obtained. One is the relatively small number of patients with esophageal‐MPTs. This prevented us to perform a detailed survival analysis in this group of patients. Another limitation is the absence of a control group of patients with HNSCC who did not develop an MPT. Consequently, we had to compare the survival of our patients with MPTs with previously reported data of patients with HNSCC without MPTs. This also prohibited us to compare the difference in effect on survival between MPTs and residual/recurrent tumors and to identify risk factors and risk profiles for the occurrence of MPTs. It could also be argued to exclude patients with tumors with a known low risk to develop multiple primary tumors (HPV‐negative oropharyngeal and sinonasal tumors). However, they were a minority of our total study population. Another point of concern is the fact that the distinction between a lung‐MPT and a distant lung metastasis is challenging—ideally, identification of genetic relation between both tumors. In this study, loss‐of‐heterozygosity analysis was performed in most cases of lung cancer. However, some exceptions were made for patients with (a) lung tumors that developed >5 years after the index HNSCC tumor and (b) patients who were treated with a palliative intent because of stage IV lung tumors. Despite these limitations, our protocolled method of data collection and large total cohort size made it possible to draw reliable conclusions from our results.

In conclusion, about 1 in 10 patients with HNSCC developed MPTs in the HN‐region, lung, or esophagus. This could be explained by the FC theory. MPTs had a negative effect on the survival, which was most pronounced in patients with MPTs which were synchronous or in the lung or esophagus. Patients with metachronous MPT in the HN‐region had a surprisingly good 5‐year survival rate. Screening and a better follow‐up might be considered to increase the overall survival of patients with HNSCC because of the high incidence and the negative effect on survival. This specifically applies to MPTs that develop in the lung and esophagus. Future goals of research are to compare patients with HNSCC with and without MPTs and identify risk factors and risk profiles for their development.

## FUNDING INFORMATION

This research did not receive any specific grant from funding agencies in the public, commercial, or not‐for‐profit sectors.
